# The FtcR-Like Protein ActR in *Azorhizobium caulinodans* ORS571 Is Involved in Bacterial Motility and Symbiosis With the Host Plant

**DOI:** 10.3389/fmicb.2021.744268

**Published:** 2021-11-19

**Authors:** Li Sun, Dandan Wang, Zhiqiu Yin, Chengsheng Zhang, Amber Bible, Zhihong Xie

**Affiliations:** ^1^National Engineering Laboratory for Efficient Utilization of Soil and Fertilizer Resources, College of Resources and Environment of Shandong Agricultural University, Taian, China; ^2^Marine Agriculture Research Center, Tobacco Research Institute of Chinese Academy of Agricultural Sciences, Qingdao, China; ^3^Oak Ridge National Laboratory, Biosciences Division, Oak Ridge, TN, United States; ^4^Key Laboratory of Coastal Environmental Processes and Ecological Remediation, Yantai Institute of Coastal Zone Research, Chinese Academy of Sciences, Yantai, China

**Keywords:** *Azorhizobium caulinodans* ORS571, transcriptional regulator, flagella, exopolysaccharide, biofilm, symbiosis

## Abstract

Bacterial signal transduction pathways are important for a variety of adaptive responses to environment, such as two-component systems (TCSs). In this paper, we reported the characterization of a transcriptional regulator in *Azorhizobium caulinodans* ORS571, ActR, with an N-terminal receiver domain and one C-terminal OmpR/PhoB-type DNA binding domain. Sequence analysis showed that ActR shared a high similarity with FtcR regulator of *Brucella melitensis* 16M known to be involved in flagellar regulation. The structural gene of this regulator was largely distributed in Alphaproteobacteria, in particular in Rhizobiales and Rhodobacterales, and was located within clusters of genes related to motility functions. Furthermore, we studied the biological function of ActR in *A. caulinodans* grown at the free-living state or in association with *Sesbania rostrata* by constructing *actR* gene deletion mutant. In the free-living state, the bacterial flagellum and motility ability were entirely deleted, the expression of flagellar genes was downregulated; and the exopolysaccharide production, biofilm formation, and cell flocculation decreased significantly compared with those of the wild-type strain. In the symbiotic state, Δ*actR* mutant strain showed weakly competitive colonization and nodulation on the host plant. These results illustrated that FtcR-like regulator in *A. caulinodans* is involved in flagellar biosynthesis and provide bacteria with an effective competitive nodulation for symbiosis. These findings improved our knowledge of FtcR-like transcriptional regulator in *A. caulinodans*.

## Introduction

Legumes obtain nitrogen through rhizobia residing in root nodules. *Sesbania rostrata*, one of the most valuable green manure legumes, could grow rapidly in waterlogged conditions and represent a high rate of nitrogen fixation. *Azorhizobium caulinodans* establishes a specific symbiosis with *S. rostrata*, which specially induces effective nodules on the stems of *S. rostrata* besides root nodulation. It also could infect cereal crop wheat (*Triticum aestivum* L.) and form para-nodules to provide 16–23% nitrogen for host wheat (Kennedy et al., 1997). What makes it more unique is that *A. caulinodans* could fix N_2_ in the free-living state (outside of the nodule) (Dreyfus et al., 1983). The wide adaptability and applicability in agriculture of *A. caulinodans* are reasons for its great attractions.

Two-component systems (TCSs) are the main paradigm for bacterial signal transduction, which are responsible for sensing and responding to a variety of physical and chemical signals (Mitrophanov and Groisman, 2008). A well-known example is one of chemotactic TCSs, CheA/Y. In *A. caulinodans* ORS571, the chemotaxis ability of *cheY* deletion mutant was defective, and its colonization on host plant was impaired (Liu et al., 2020a). TCS NtrX/Y was identified in *A. caulinodans*, which was responsible for nitrogen metabolism in free-living state and nodulation in symbiosis (Pawlowski et al., 1991). TCSs also play a crucial role in the process of flagellar synthesis and motility. For example, the TCS FleS/R was required for transcription of 20 or more flagellar biosynthetic genes in *Pseudomonas aeruginosa* (Dasgupta et al., 2003). In the monoflagellate *Shewanella oneidensis*, the TCS FlrB/C was related to regulating the expression of *flaA* and *flaB* genes related to flagellar motility (Shi et al., 2014). Bacterial flagella are complex rotary engines like a propeller embedded in the cell envelope, which participate in a variety of processes, including motility, biofilm formation (Pratt and Kolter, 2010), colonization (Shen et al., 2018), and cell invasion (Horstmann et al., 2020).

Rhizobia manifest a range of behaviors leading to symbiotic association with host plants, which mainly consist of chemotaxis and motility, adhesion, colonization, infection, and nodulation. For the mechanisms of symbiosis, we have always focused on the effect of chemotaxis and flagellar motility on symbiosis, because they are the first and key steps in establishing plant–microbe interaction (Brencic and Winans, 2005; Jiang et al., 2016; Yang et al., 2017; Liu W. et al., 2018; Shen et al., 2018; Liu et al., 2019, 2020a). The flagellar motor proteins FliM and FliN in *A. caulinodans* were involved in flagellum synthesis, bacterial motility, and biofilm formation (Shen et al., 2018). The genome of *A. caulinodans* contains the *che* (chemotaxis) gene cluster (*cheA*, *cheW*, *cheY1*, *cheB*, and *cheR*); and their relevant mutants were detected for defection in competitive colonization and nodulation with *S. rostrata* (Liu W. et al., 2018). Latterly, it was found that *cheZ*-like gene of *A. caulinodans* was a key gene in influencing root colonization by regulating exopolysaccharide (EPS) production (Liu X. et al., 2018; Liu et al., 2019). Besides *cheY1* (in the *che* gene cluster), *A. caulinodans* possesses another gene encoding CheY protein, *cheY2* (outside the *che* gene cluster). Both of them were involved in chemotaxis, while they had different regulatory mechanisms, and CheY2 had a more remarkable role (Liu et al., 2020a). The whole-genome sequence of *A. caulinodans* has been obtained (Lee et al., 2008), *AZC_0619* is closely located upstream of *cheY2* and *cheZ*, and it is a homolog of *ftcR* gene of *Brucella melitensis* 16M. FtcR, a two-component response regulator, regulated flagellar gene expression (Léonard et al., 2007). The biological functions of the *ftcR* in *B. melitensis* raise the questions of whether *ftcR*-like gene in *A. caulinodans* exhibits similar regulation in flagellar genes expression or has specific symbiotic properties.

In this study, the main aim was to elucidate the biological functions of ActR in *A. caulinodans* ORS571 under free-living and symbiotic conditions. We found that ActR protein regulated motility, EPS production, biofilm formation, cell flocculation, root colonization, and competitive nodulation on the stems of *S. rostrata*. This study provides important insights on the two-component regulatory FtcR-like protein in bacterial motility and symbiosis of ORS571.

## Materials and Methods

### Bacterial Strains, Plasmids, and Culture Conditions

All bacterial strains, plasmids, and primers used in this study are shown in Tables 1, 2. *A. caulinodans* ORS571 was grown in TY or L3 media with ampicillin and nalidixic acid at 37°C. *Escherichia coli* DH5α strain was grown in LB medium at 37°C.

**TABLE 1:** Bacterial strains and plasmids used in this study.

Strains and plasmids	Relevant characteristics^a^	Source
**Strains**		
*Azorhizobium caulinodans* ORS571	Wild-type strain, NaI^R^ and Amp^R^	Dreyfus et al., 1988
Δ*actR*	ORS571 derivative, Gen^R^	This study
Δ*actR*-C	Complemented strain of Δ*actR*	This study
WT-O	The *actR* overexpression strain	This study
*Escherichia coli* DH5α	General cloning strain	TransGen
**Plasmids**		
pEASY Blunt Simple	The vector cloning PCR products, Kan^R^	TransGen
pCM351	The construction of mutant, Gen^R^ and Tc^R^	Marx and Lidstrom, 2002
pRK2013	Helper plasmid carrying *tra* genes, Kan^R^	Figurski and Helinski, 1979
pBBR1MCS-2	Broad-host-range cloning vector, Kan^R^	Kovach et al., 1995
pBBR-*actR*	pBBR1MCS-2 with *actR* open reading frame and upstream promoter region, Kan^R^	This study

*^a^Nal^R^, Amp^R^, Gen^R^, Kan^R^, and Tc^R^ represent nalidixic acid resistance, ampicillin resistance, gentamicin resistance, tetracycline resistance, and kanamycin resistance, respectively.*

**TABLE 2:** Primers used in this study.

Primer	Sequences (5′–3′)	Restriction site
*actR*-up-F	GGTACCACTTCCTTCAGAAGCCGCT	*Kpn* I
*actR*-up-R	CATATGGAAGCTGCGCATGAAGCTC	*Nde* I
*actR*-down-F	ACCGGTAGCTGGAAACGTAACCGTC	*Age* I
*actR*-down-R	GAGCTCCTCCTTCAGATCCGTTCAG	*Sac* I
*actR* in-F	CATGCGCAGCTTCTTGCGC	
*actR* in-R	AGGACATCGGTGCCGTGCA	
*actR-Xho*I-F	CCCTCGAGACATCCTGAACGGTAGAGGAAC	*Xho* I
*actR-Bam*HI-R	CGGGATCCTCGTCCTCTTGCACCGTCACGAAC	*Bam*H I
*motB*-F	ATCGCTCCGACACCTATGAC	
*motB*-R	TCCGCCTTGTTCTTCAGATT	
*fliF*-F	GCGAAGAGCTCACCAATTTC	
*fliF*-R	GCTCGATCTCGTAGGTCTGG	
*fliL*-F	CTTGCCATCCTGATCCTCAC	
*fliL*-R	AGCTGCTGCTCCTTCTTCAC	
*flg*I-F	ACAACCAGCTCATCGGCTAC	
*flgI*-R	TGTTGAGCTGCGTGATAAGC	
*flgG*-F	GCTCGGCAACAACCTCTATC	
*flg*G-R	ACGGTCTTCGACATTTCGTC	
*flhB*-F	CCAAGCAGGAAGTGAAGGAG	
*flhB*-R	CCCTTCTGGCGGTCATAATA	
*fliG*-F	AGGGCCTGCTCTTTACCTTC	
*fliG*-R	GTCGTTGCCGGTATTGATCT	
*fliM*-F	CTCGAGGAGCGACACTATCC	
*fliM*-R	GTCATCGACGAACTCCTGGT	

Antibiotics were used as follows: nalidixic acid (Nal), 25 μg/ml; kanamycin (Kan), 30 μg/ml; gentamicin (Gen), 30 μg/ml; tetracycline (Tc), 10 μg/ml; and ampicillin (Amp), 100 μg/ml. TY medium contains tryptone (5 g/L), yeast extract (3 g/L), and CaCl_2_ (0.6 g/L). The L3 medium contains KH_2_PO_4_ (1.36 mg/L), NH_4_Cl (0.53 mg/L), carbon source (adjusting the carbon according to the need, 10 mM), MgSO_4_ (100 mg/L), NaCl (50 mg/L), CaCl_2_ (40 mg/L), FeCl_3_ (5.4 mg/L), Na_2_MoO_4_ (5 mg/L), biotin (2 mg/L), nicotinic acid (4 mg/L), and pantothenic (4 mg/L). LB medium contains tryptone (10 g/L), yeast extract (5 g/L), and NaCl (10 g/L).

### Sequence Analysis

The domains of ActR (AZC_0619) were predicted by InterPro annotation (Mitchell et al., 2019). The Kyoto Encyclopedia of Genes and Genomes (KEGG) database was searched for the protein sequences of FtcR-like proteins (accession number: K21603) (Kanehisa et al., 2021). Alignments of the protein sequences of FtcR-like protein were done using COBALT with the default parameters (Papadopoulos and Agarwala, 2007). The maximum likelihood (ML) tree of FtcR-like protein sequences was reconstructed using the MEGAX with the LG amino acid substitution model and gamma distributed with invariant sites (G+I) (Kumar et al., 2018). Furthermore, complete deletion of gaps and missing data was carried out to exclude highly variable regions. The AZC_0619 and selected proteins were shown in a logo that was generated by Weblogo with the default parameters (Crooks et al., 2004) and further analyzed with ESPript 3.0 (Robert and Gouet, 2014).

### Construction of the Deletion Mutant and Complemented Strain

Genomic DNA was extracted from *A. caulinodans* ORS571, and the *actR* deletion mutant was constructed using a methodology of allelic exchange mutagenesis. First, fragments of upstream and downstream genes of *actR* were amplified from the genomic DNA of *A. caulinodans* ORS571 with two pairs of primers (*actR*-up-F and *actR*-up-R, and *actR*-down-F and *actR*-down-R), carrying *Kpn*I, *Nde*I, *Age*I, and *Sac*I restriction enzyme sites, respectively (Table 1). These fragments were cloned into vector pEASY-Blunt Simple to generate plasmid pEASY::*actR* up and pEASY::*actR* down and then, respectively, linked with pCM351 to generate pCM351::*actR* up–down by restriction enzyme digestion. Then, pCM351::*actR* up–down was transformed into *E. coli* DH5α competent cells. Finally, pCM351::*actR* up–down was introduced into *A. caulinodans* ORS571 via allelic exchange, and *actR* gene was replaced. Mutations were selected in TY medium with Nal, Amp, and Gen and were verified by PCR using a pair of primers: *actR*-up-F and *actR*-down-R. The mutant with a deleted *actR* gene was named Δ*actR*.

To construct the complemented strain of Δ*actR* (Δ*actR*-C), the fragment of the entire open reading frame and predicted promoter of *actR* was amplified, then was digested with *Xho* I and *Bam*H I, and cloned into plasmid pBBR1MCS-2 (Kovach et al., 1995). The recombinant plasmid pBBR-*actR* was introduced into Δ*actR* mutant by triparental conjugation. The complemented strain was named as Δ*actR*-C. The ActR overexpressed strain, Δ*actR* with empty plasmid pBBR1MCS-2, and wild type (WT) with empty plasmid pBBR1MCS-2 were constructed in the same way and was designated as WT-O, Δ*actR*-P, and WT-P, respectively.

### The Growth Curve

WT and Δ*actR* mutant strains were cultured overnight with TY medium containing antibiotics. The overnight cultures were collected and adjusted to an optical density (OD) at OD_600_ of 0.05. Normalized cultures measuring 500 μl of the WT and mutant were inoculated into 50 ml of TY medium and then were cultured in a rotary shaker at 37°C. The value of OD_600_ was measured every 2 h. The data were recorded as means and SDs from three repetitions.

### Swimming Assay

The swimming assay was analyzed on L3 soft agar (0.3% agar) plates containing succinate as the sole carbon source and 10 mM of NH_4_Cl. Overnight cultures were adjusted to a density at OD_600_ of 1.0. Aliquots measuring 5 μl of cell suspensions of the WT, Δ*actR*, Δ*actR*-C, Δ*actR*-P, WT-P, and WT-O were inoculated on L3 soft agar plates for 48 h at 37°C.

### Transmission Electron Microscopy

The bacterial strains were cultured in TY medium for 24 h at 37°C. The overnight cultures were collected and washed twice with sterile phosphate-buffered saline (PBS), spotted on copper grids, and negatively stained with 2% phosphotungstic acid. The transmission electron microscopy (TEM) images of the WT and Δ*actR* mutant were taken at random with grid.

### Quantitative Real-Time PCR

The total RNA was isolated from *A. caulinodans* ORS571 using TransZol Up Plus RNA kit. cDNA was generated through TransScript One-Step gDNA removal kit according to the manufacturer’s instructions. The synthesized cDNA was diluted 500-fold and used as template to analyze relative gene expression. The quantitative qPCR was performed with GoTaq^®^ qPCR Master Mix kit using gene specific primer pairs (Table 2). The quantitative PCR program consisted of an initial denaturation at 95°C for 2 min, followed by 40 cycles of 95°C for 15 s, 60°C for 30 s, and 72°C for 1 min. To evaluate the gene expression, the copy number of each gene was normalized to that of the 16S rRNA. The analysis of its results was performed using the comparative cycle threshold method (Schmittgen and Livak, 2008).

### Biofilm Formation Assay

WT and Δ*actR* mutant were incubated overnight in the L3 medium at 37°C, and then the culture was adjusted to OD_600_ of 2. Bacterial suspensions measuring 15 μl were added into glass tubes including 1.5 ml of L3 medium and were incubated for 3 days at 37°C. The L3 medium contained succinic acid (10 mM) as a sole carbon source, with nitrogen. After 3 days’ incubation, the glass tubes were gently washed three times with PBS (pH = 7.2) to remove free-floating bacteria and then stained with 2 ml of crystal violet (CV; 0.1%, w/v) for 20 min. CV was gently removed by washing three times with deionized water. Finally, biofilm formation was quantified by ethanol-solubilized CV from glass tube biofilms. The OD value of each tube was determined after CV staining with a wavelength of 590 nm.

### Exopolysaccharide Production

EPS production was estimated using the method described by Jiang et al. (2016) with the following modifications. The WT and Δ*actR* mutant were incubated in the L3 medium at 37°C. Then, bacterial cultures were adjusted to an OD_600_ of 0.8. Ten-microliter aliquots of bacterial suspensions were spotted on L3 solid agar (0.8% agar) plates with three different carbon sources (sodium lactate, glycerol, and malic acid) and incubated at 37°C. The L3 plates included 10 mM of carbon source, 10 mM of NH_4_Cl, and 40 μg/ml of Congo red. Photographs were taken after 4 days of incubation. The quantitative analysis of EPS content was measured by a colorimetric method using anthrone and sulfuric acid and was evaluated by normalizing to OD_600_ of the bacterial suspension.

### Flocculation

Flocculation was measured using the method described by Jiang et al. (2016). Overnight cultures were normalized to an OD_600_ of 1.0, and then 200 μl of the normalized cultures was inoculated into 10 ml of L3 medium containing 10 mM of sodium lactate as the carbon source and 0.5 mM of NH_4_Cl as the nitrogen source. Photographs of the non-flocculated cells and flocculated cells were taken after 48 h of incubation. For the quantitative analysis, the bacterial suspensions were left to stand for 30 min. The non-flocculated cells were removed from the tube, and the OD_600_ of the suspension (ODs) was measured. The flocculated cells that settled to the bottom of the tube were dispersed using a tissue homogenizer and thoroughly mixed with free cells; the density of the mixed culture was also measured at 600 nm and named as OD_t_. The percent flocculation was calculated as follows: percent flocculation = (1 – OD_s_/OD_t_) × 100.

### Rhizosphere Colonization Assay

Seeds of *S. rostrata* were treated with concentrated sulfuric acid for 30 min, followed by rinsing five times with sterilized water. Sterile seedlings were germinated in the dark at 37°C for 72 h. WT and mutant strains were grown overnight in sterile TY medium; then overnight cultures were collected and adjusted to a density of 0.8. For competitive colonization, suspensions of the WT and mutant were mixed at 1:1, 1:5, and 1:10. Germinated *S. rostrata* seeds were inoculated with the mixed cultures for 1 h. The surfaces of seedlings were washed seven times using sterilized water. And then the root tips were vortexed, and bacteria were reisolated on TY agar plates with ampicillin. A total of 100 of the colony-forming units (CFUs) were selected, and the number of the WT and Δ*actR* was counted by plate streaking in TY agar plates with ampicillin and gentamicin. In addition, the WT and mutant colonies were also identified by PCR.

### Competitive Nodulation Assay

Surface-sterilized seedlings of *S. rostrata* were grown in soil supplemented with sterilized vermiculite and low nitrogen plant nutrient solution for 4 weeks. The bacterial suspension (OD_600_ = 0.6) of the WT and Δ*actR* mutant strains was mixed at ratios of 1:1 and 1:5; then the mixed cultures were used to paint the stem surface of *S. rostrata*. After 30 days’ inoculation at 27°C in the greenhouse, nodules were harvested, and bacteria were reisolated and plated on TY agar medium from stem nodules. The WT and mutant bacteria were identified and distinguished by PCR using the primer pair *actR* in-F and *actR* in-R, and the ratios between the WT and mutant were further counted.

### Statistical Analysis

Mean and standard errors were measured based on each experiment repeated three times. The Statistical Package for the Social Sciences (SPSS version 20) was used to analyze the least significant difference test (*p* < 0.05).

## Results

### The Genome of *Azorhizobium caulinodans* Encodes a Homolog of the Motility Regulator FtcR

**FIGURE 1:**
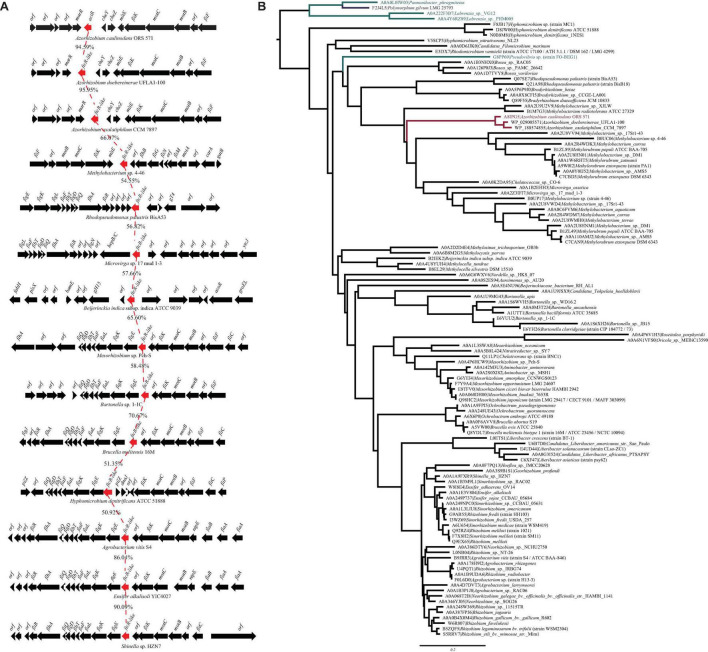
**(A)** Genetic organization of gene clusters in the genomic region containing the *ftcR*. The *frcR* coding sequences (CDS) is indicated in red. Percentage amino acid identities of the FtcR are shown. **(B)** Maximum likelihood phylogeny of the protein sequences of FtcR homologs. The clades of the order Rhizobiales, Rhodobacterales, and Polymorphum are indicated in black, turquoise, and blue, respectively. The *Azorhizobium* clade and A8IPG5 (ActR; *Azorhizobium caulinodans* ORS571) are indicated in red.

From the genome sequence of *A. caulinodans* ORS571, we searched for the presence of FtcR homolog, AZC_0619. Only one open reading frame encoding for the FtcR-like protein was identified, which was named as ActR. Sequence analysis showed that ActR contains 222 amino acids, encoding one N-terminal receiver domain (IPR001789) and one C-terminal OmpR/PhoB-type DNA binding domain (IPR001867) (Supplementary Figure 1). A BLASTp analysis exhibited that AZC_0619 shared about 53.2% of identity with a flagellar master regulator FtcR of *B. melitensis* 16M. *actR* gene was located downstream of two chemotactic genes (*cheY* and *cheZ*), *mltE* gene encoded for transmembrane protein, and three flagellar genes (*motB*, *motC*, and *fliK*) in *A. caulinodans* (Figure 1A). And flagellar genes were found in the proximity of *ftcR*-like gene in most species (Figure 1A), indicating that FtcR-like protein may play a role in the regulating flagellar motility among Rhizobiales.

To clarify the evolutionary relationships of the FtcR-like proteins, a phylogenetic tree was generated. As shown in Figure 1B, the FtcR-like tree exhibited extensive genetic diversity. FtcR-like proteins among Rhizobiales members were monophyletic and exhibited topology that was generally congruent with the species taxonomy [KEGG Organisms in the National Center for Biotechnology Information (NCBI) Taxonomy], suggesting that FtcR-like proteins originated from a last common Rhizobiales ancestor and underwent divergent evolution during species differentiation. Within this phylogenetic tree, we found that FtcR-like proteins in *Azorhizobium* formed a monophyletic subclade with long branch lengths (Figure 1B). Notably, FtcR-like proteins from *A. caulinodans* ORS571 and *B. melitensis* 16M are clustered into separated clades.

**FIGURE 2:**
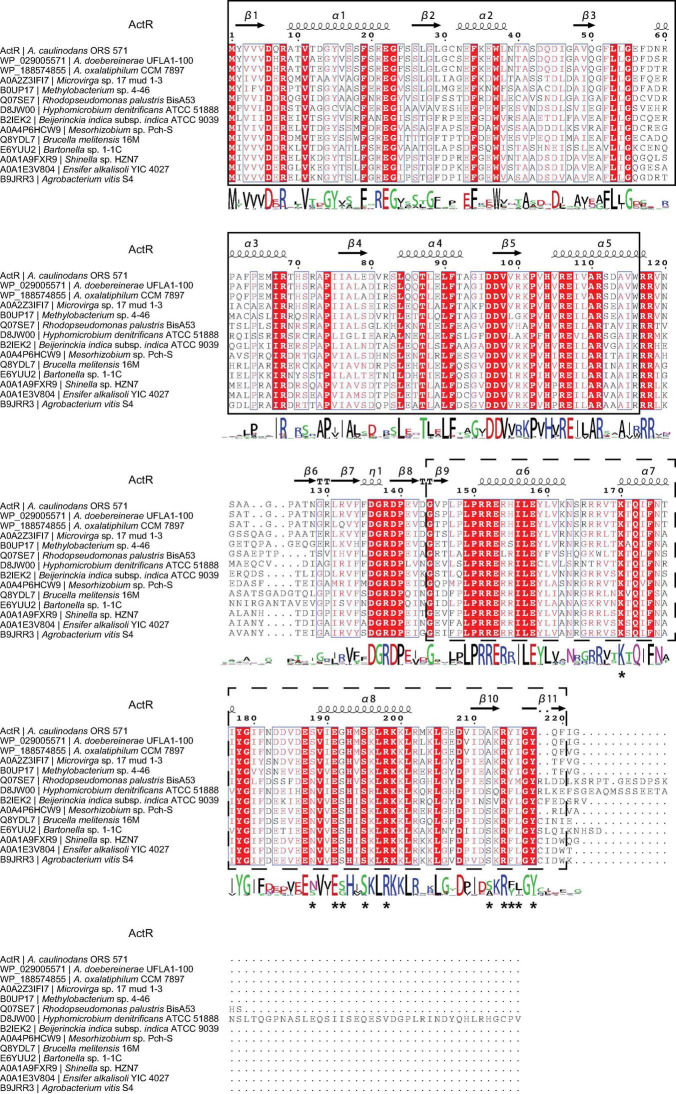
Analysis of the FtcR-like sequence. Protein sequences were aligned using the COBALT with the default parameters. The picture of the alignment was performed by ESPript 3.0. The predicted secondary structure of ActR is shown above the alignment. Conserved residues are shown with red background, and similar residues are shown in red and boxed. The sequence logo is shown below the alignment. The bigger the logo, the more conserved the residue. The ligand binding site residues of ActR predicted by the COFACTOR and COACH programs in I-TASSER are marked with asterisks. The solid line frame and the dashed line frame indicate the response regulator domain and the transcriptional regulatory domain (OmpR/PhoB-type) as recognized in InterPro annotation.

We then systematically examined the prevalence and evolution of FtcR. Based on the KEGG orthology database (KO: K21603) (Kanehisa et al., 2021), FtcR-like proteins were present in orders Rhizobiales, Rhodobacterales, and Polymorphum of Alphaproteobacteria. FtcR-like proteins were widespread in most genus/species among Rhizobiales and had a sporadic distribution among Rhodobacterales (genera *Pseudovibrio*, *Pannonibacter*, and *Labrenzia*). A total of 117 protein sequences of FtcR-like were recovered from the KEGG database (KO: K21603) and exhibited a significant divergence (more than 35.4% identity) at the amino acid level (Figure 2). The FtcR-type sequences are conserved in genus *Azorhizobium* (*A. caulinodans*, *Azorhizobium doebereinerae*, and *Azorhizobium oxalatiphilum*) with the pairwise sequence identities in the range of 93.24–95.59% (Figures 1A, 2); this result was consistent with that of *Azorhizobium* FtcR proteins that formed a monophyletic subclade in Figure 1B. Our analysis indicated that FtcR-like protein is conserved and that it may play an important role among *Azorhizobium*. Although Léonard et al. (2007) provided insights on the role of flagellar master regulator FtcR in *Brucella*, the function of its homology in rhizobia has remained unclear. Therefore, we will investigate the biological function of ActR in *A. caulinodans* ORS571.

### ActR Regulates Cell Motility and the Expression of Flagellar Genes

**FIGURE 3:**
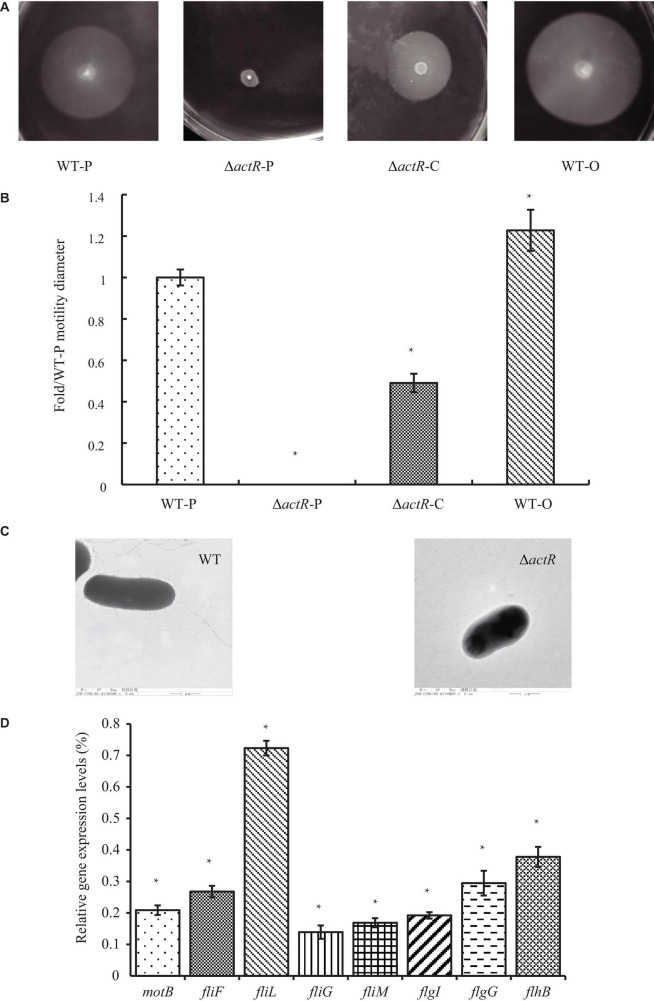
ActR was required for swimming motility and flagellar biosynthesis in *Azorhizobium caulinodans*. **(A)** Representative images of the bacterial motility rings in soft agar plates, including the wild-type (WT) strain (WT-P), the Δ*actR* strain (Δ*actR*-P), the complemented strain (Δ*actR*-C), and the *actR*-overexpressing strain (WT-O). **(B)** Quantitative analysis of the swimming motility abilities. Bar plot represents the mean and SD of fold change about the bacterial motility ring diameter compared with that of WT-P. **(C)** Representative images obtained by transmission electron microscopy using cells from the WT and Δ*actR* strains. Scale bar represents 1 μm. **(D)** Relative expression of flagellar genes (*motB*, *fliF*, *fliL*, *fliG*, *fliM*, *flgI*, *flgG*, and *flhB*) in the Δ*actR* mutant strain compared with that of WT. Bar plot represents the mean and SD of the gene expression level. Means and SDs of fold change and gene expression were obtained from at least three independent experiments. Student’s *t*-test; *represents significant difference at *p* < 0.05.

ActR was predicted to be a flagellar two-component response regulator. To characterize the role of ActR in regulating motility, Δ*actR* mutant strain was constructed through allelic exchange mutagenesis (see section “Materials and Methods,” Supplementary Figure 2), and the swimming motility behavior of the Δ*actR* mutant and complementary strain were compared with those of the WT (Figures 3A,B). We found that the mutant (Δ*actR-*P) with an empty plasmid pBBR1MCS-2 was devoid of motility ability, which could be rescued by introducing the pBBR1MCS-2 carrying WT *actR* gene and its native promoter into the mutant (Δ*actR-*C) (Figure 3A). We also found that an overexpressing strain (WT-O) exhibited significant increase of swimming motility when compared with that of the WT-P. The WT-O and WT-P were constructed by the introduction of an empty plasmid pBBR1MCS-2 and a pBBR1MCS-2 carrying WT *actR* gene and its native promoter, respectively. The quantitative data clearly confirmed the result, too (Figure 3B).

To explore the reason for lost motility ability, it was first checked that growth properties of the mutant strain was not impaired (Supplementary Figure 3). What is really interesting was that TEM of the WT and the mutant strains revealed the absence of flagella in the mutant strain (Figure 3C).

Next, we tested whether ActR regulator regulated the expression of flagellar synthesis-related genes by quantitative real-time PCR. It is true that the deletion of any flagellar component is sufficient to abolish flagellar synthesis, such as FliM and FliN (Shen et al., 2018). The results showed that the expression levels of eight flagellar genes (*motB*, *fliF*, *fliL*, *fliG*, *fliM*, *flgI*, *flgG*, and *flhB*) in Δ*actR* mutant were significantly downregulated than those in the WT (Figure 3D). For example, the expression values of *fliL* and *fliG* declined 27 and 88%, respectively. These results suggested that ActR was closely associated with the synthesis of flagella and positively regulated the motility of *A. caulinodans* ORS571.

### ActR Positively Regulates Biofilm Formation and Exopolysaccharide Production

**FIGURE 4:**
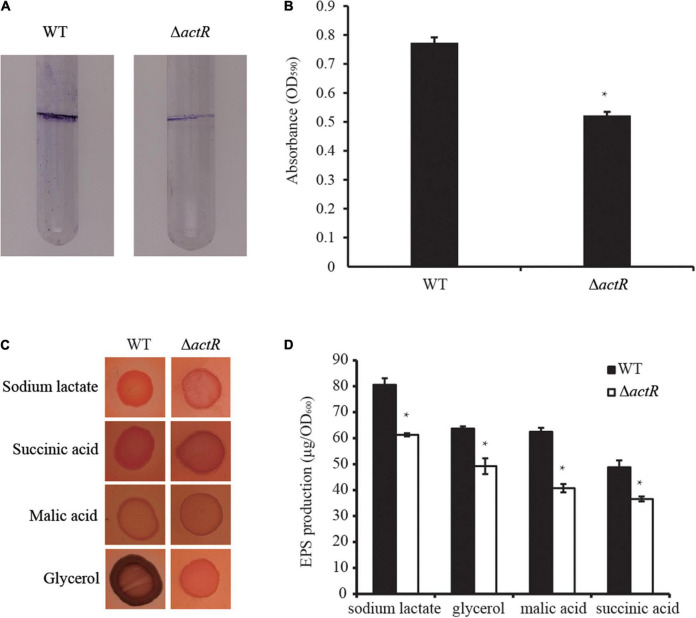
Effects of ActR on the biofilm formation and exopolysaccharide (EPS) production of *Azorhizobium caulinodans* ORS571. **(A)** Representative images of biofilms formed by the wild type (WT) and Δ*actR.* **(B)** Quantitative analysis of biofilm formation. Bar plot represents the mean and SD of the optical density at 590 nm. **(C)** Representative images of colony morphologies of the WT strain and Δ*actR* mutant spotted on the L3 plates with Congo red staining and four different carbon sources (sodium lactate, glycerol, malic acid, and succinic acid). Photographs were taken after 4 days of incubation. **(D)** Quantitative analysis of the EPS production. Bar plot represents the mean and SD of the optical density at 600 nm of EPS production. Means and SDs of biofilm formation and EPS production were obtained from at least three independent experiments. Student’s *t*-test; *represents significant difference at *p* < 0.05.

Biofilm formation and motility are modulated directly by flagellar power (Subramanian and Kearns, 2019), and biofilm forming on plant surfaces is important for bacterial symbiotic interaction with host plant (Xu et al., 2019). To investigate whether the deletion of *actR* has an influence on bacterial biofilm formation, we tested the biofilm of the WT and the mutant. As shown in Figure 4A, the mutant produced less biofilm than the WT in the L3 medium with nitrogen (L3+N medium), and quantitative data confirmed the results. In the L3+N medium, the biomass of biofilm in Δ*actR* mutant was 32% less than that of the WT strain (Figure 4B).

We next tested the production of EPS by Congo red staining. EPS is essential for the maintenance of biofilm formation in bacteria (Cugini et al., 2019). And it is involved in bacterial symbiotic nodulation with *S. rostrata* (Sun et al., 2020). To study the EPS production ability of Δ*actR*, the colony morphologies and quantitative analysis of the EPS were examined using minimal medium plates containing different carbon sources (sodium lactate, glycerol, malic acid, and succinic acid). A significant difference between the WT and the mutant was observed. The colonies of the WT cells produced more EPS than Δ*actR* cells regardless of the kind of carbon sources were assayed (Figures 4C,D). These data indicated that ActR positively affected EPS production and biofilm formation in *A. caulinodans* ORS571.

### ActR Contributes to Cell Flocculation

**FIGURE 5:**
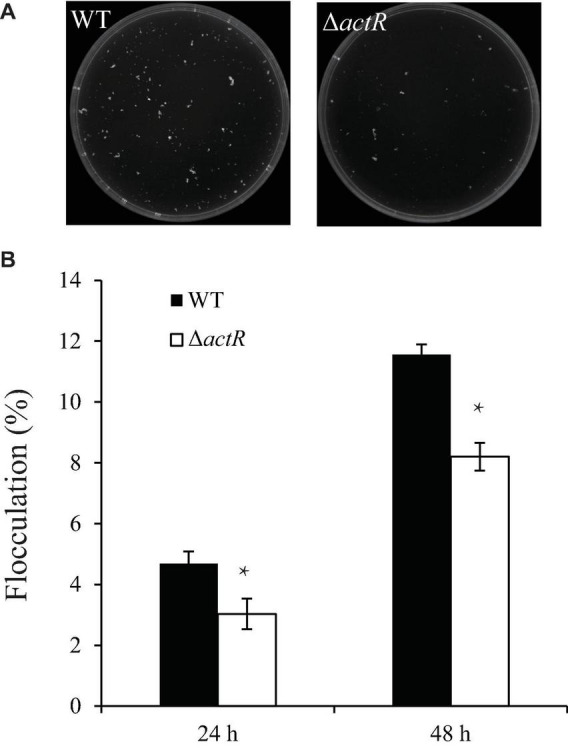
Effect of ActR on the flocculation generated by wild-type (WT) and mutant strains. **(A)** Representative images of flocculation morphologies produced by the WT and Δ*actR* mutant. Strains were inoculated with L3+1/2N liquid medium, and photographs were taken after 48 h of incubation. **(B)** Quantitative analysis of the flocculation cell mass. Bar plot represents the mean and SD of the percent density of flocculation generated by each strain. Means and SDs were obtained from at least three independent experiments. Student’s *t*-test; *represents significant difference at *p* < 0.05.

Flocculating substances are secreted by many microorganisms in the culture broth, which comprise polysaccharides, proteins, and lipids (Salehizadeh and Yan, 2014). To study whether ActR influences flocculation in *A. caulinodans* ORS571, we tested the flocculation morphologies between the WT and mutant strains in the L3 medium with 5 mM of NH_4_Cl as a nitrogen source. Figure 5A shows that flocs formed by the WT were larger and more abundant than those of the mutant strain. Quantitative analysis suggested that the WT flocculated more than the mutant strain and that the flocculation formation of both strains increased over time (Figure 5B). These results indicated that ActR was involved in regulating flocculation formation.

### ActR Is Involved in Root Colonization and Nodule Formation by *Azorhizobium caulinodans* ORS571

**FIGURE 6:**
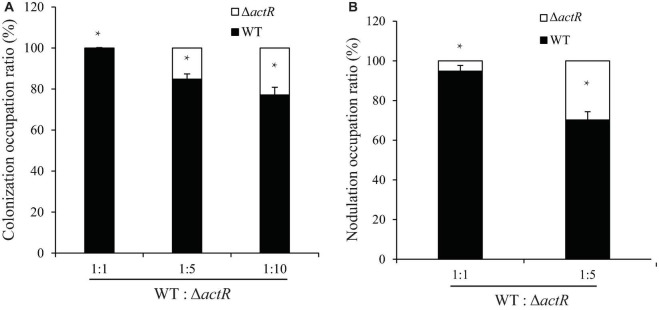
The mutant reduced colonization and nodulation competitiveness on *Sesbania rostrata.* **(A)** Competitive colonization of wild type (WT) and Δ*actR* mutant strains on *S. rostrata* root. Ratios of 1:1, 1:5, and 1:10 represent the ratio of WT to the mutant. **(B)** Competitive nodulation of WT and Δ*actR* on the stems of *S. rostrata*. Ratios of 1:1 and 1:5 represent the WT to the mutant. Bar plot represents the mean and SD of the relative colonization ratio and the relative nodulation occupation ratio of the WT (black bar) and Δ*actR* mutant (white bar). Means and SDs were obtained from at least three independent experiments. Student’s *t*-test; *represents significant difference at *p* < 0.05.

The colonization on the surface of the host plant is a key step for successfully establishing symbiosis. To investigate the symbiotic role of ActR in *A. caulinodans*, competitive colonization experiments were performed. We counted the number of cells reisolated from the seedlings to verify the efficiency of competitive colonization. As shown in Figure 6A, the Δ*actR* mutant was less competitive than the WT. When the WT and the mutant cells were mixed in equal proportion, 100% of the bacteria reisolated from the root surface belonged to the WT, showing that the mutant could not compete. When the proportions between the WT and mutant were 1:5 and 1:10, a few of the cells of Δ*actR* mutant strains (14.3 and 23.1%, respectively) were reisolated from the root system. This result suggested that the deletion of *actR* did not disable bacterial colonization ability but affected *A. caulinodans* with an effective competitive ability for root colonization.

Induction of nodule morphogenesis is associated with the EPS production and colonization of the root system (Liu and Xie, 2019). To study whether ActR plays an important role in nodule formation, the competitive nodulation assay was performed by counting the number of cells (WT and mutant strains) reisolated from the stem nodules. As shown in Figure 6B, the ratios of Δ*actR* mutant were 8 and 31% when the WT and mutant strains were mixed at ratios of 1:1 and 1:5, respectively, indicating that the Δ*actR* mutant could still nodulate *S. rostrata* but not compete with the WT strain. Taken together, these results indicated that ActR could provide *A. caulinodans* an effectively competitive ability for symbiosis.

## Discussion

The FtcR-like regulator is widely distributed across genus/species within Rhizobiales, such as *A. caulinodans*, *Ensifer meliloti*, and *Agrobacterium rhizogenes*. The phylogeny general congruence with species taxonomy and sequence divergence between homologs indicated that FtcR-like proteins in Rhizobiales were predominantly vertically inherited from a common ancestor and underwent divergent evolution during species differentiation. In this study, *AZC_0619* in *A. caulinodans* (named *actR*) was characterized, which was a homolog of *ftcR* gene of *B. melitensis* 16M. This gene is generally in close proximity to chemotaxis and flagellum-related genes, indicating that ActR protein may play an important role in motility and symbiosis with the host plant. By the construction of Δ*actR* mutant, we found that mutant strain had no flagella and was devoid of motility. The improperly formed flagellum is a major cause of defective motility. The structure of a flagellum is constructed by a highly ordered process and consisted of about 30 flagellar proteins upon various stoichiometries (Altegoer and Bange, 2015). The flagellar gene *fliM* deletion mutant of *A. caulinodans* was confirmed to have no flagellum (Shen et al., 2018). RT-PCR revealed that the genes (*motB*, *fliF*, *fliL*, *fliG*, *fliM*, *flgI*, *flgG*, and *flhB*) involved in flagellar synthesis were downregulated in the Δ*actR* mutant. Therefore, ActR regulator in *A. caulinodans* ORS571 was involved in the flagellar assembly and further affected bacterial motility.

ActR protein in *A. caulinodans* was involved in bacterial symbiosis with the host plant. The Δ*actR* mutant still formed colonization and nodulation on *S. rostrata*, but its competitiveness was obviously eroding, which is to say that ActR hardly affected the formation of nodules, but what is the reason for the competitive disadvantage of this mutant? There are two possible explanations: first, the colonization and nodulation are related to flagella and motility. Bacterial flagella could function as adhesion molecules (Friedlander et al., 2015), and non-mobile mutant could not move away from the initial inoculation region (Liu et al., 2019). So it was unsurprising that non-flagellated Δ*actR* mutant had narrower colonization region and lower nodulation than the WT strain. It was confirmed that the impaired motility of Δ*aclR1* and Δ*acfR* mutants was consistent with the weak nodulation abilities in *A. caulinodans* (Liu et al., 2020b,^2021^). Second, EPS production and biofilm formation influence root colonization. Previous studies indicated that EPS and biofilm were essential for attachment to the root surface (Ma et al., 2006; Al-Ali et al., 2018). When the EPS biosynthesis genes of *Rhizobium* sp. IRBG74 were disrupted, the mutant exhibited defective colonization and nodulation (Mitra et al., 2016). Take the case of *A. caulinodans*: the *oac2* gene deletion mutant had decreased the EPS production ability, and its symbiosis with host plant was disabled (Gao et al., 2001). It was also proposed that EPS production regulated bacterial early colonization and the *A. caulinodans-S. rostrata* nodulation process (Liu et al., 2019). In this study, it was showed that the biofilm formation and EPS production of Δ*actR* mutant was decreased (Figure 4). Therefore, we conjectured that ActR probably helped cells in forming a symbiotic association with host plant by modulating EPS production and biofilm formation in Rhizobiales.

Based on the observation of non-flagellated morphology and decreased EPS formation of Δ*actR* mutant (Figures 3C, 5), two hypotheses about the decrease of biofilm formation were made. First, Δ*actR* mutant reduced biofilm formation due to the lack of flagella. Flagella are a component of the biofilm matrix and are vital in stabilizing biofilm by acting as bacterial biofilm scaffolds (Hathroubi et al., 2018). The non-flagellated mutants (Δ*fliM* and Δ*fliN*) in *A. caulinodans* showed the decrease of biofilm biomass compared with the wild-type strain (Shen et al., 2018). Liu et al. (2020) further verified that Δ*fliN* mutant reduced the biofilm formation after 12 h. Second, the EPS production of Δ*actR* mutant may affect biofilm formation. EPS in biofilm has vital role in maintaining the biofilm structure and providing biofilm cells with nutrients (Altaf and Ahmad, 2016). The *actR* gene deletion mutant had less EPS production regardless of what carbon source was used, which may be another reason for the decrease of biofilm formation. The positive correlation between biofilm formation and EPS was shown in Δ*cheZ* mutant of *A. caulinodans* (Liu X. et al., 2018). Meanwhile, we found that Δ*actR* mutant formed less flocculation than the WT. Flocs was encysting bacteria surrounded by EPS (Sadasivan and Neyra, 1985). In *A. caulinodans*, Sun et al. (2020) indicated that the increase of flocculation was correlated with the increase of EPS production. So we proposed that the decrease of EPS production contributed to less formation of biofilm and flocculation. As for the decrease of EPS content in Δ*actR* mutant, the possible reason is that the genes related to EPS production in Δ*actR* mutant strain did not express like that in the WT strain. Lauriano et al. (2004) reported that sodium-driven motors (*mot*), such as *motA*, *motB*, *motX*, and *motY*, were involved in EPS production, and any of the *mot* gene deletion mutants of *flaA* MO10 strain reduced EPS expression. It was observed that *motB* gene was downregulated in the Δ*actR* mutant, so flagellar motors may play a similar role in regulating EPS expression. In addition, Belas (2014) also described that EPS production was involved in the stimulation of mechanosensing signals caused by flagellar rotation. A non-flagellated Δ*actR* mutant might decrease EPS expression by reducing signal stimulation of flagellar rotation. On the other hand, ActR of *A. caulinodans* may have a specific role, not like known regulatory mechanisms of EPS expression. There is little evidence that FtcR-like protein regulates EPS production and biofilm formation in other bacteria; thus, the regulatory mechanisms of ActR to EPS production at the molecular level need to be verified further.

This study further deepens our understanding of the role of OmpR domain-containing transcriptional regulators in *A. caulinodans*. ActR not only can regulate bacterial motility but also can influence bacterial symbiosis with *S. rostrata*. However, the specific regulatory mechanism of ActR needs to be further studied.

## Data Availability Statement

The original contributions presented in the study are included in the article/Supplementary Material, further inquiries can be directed to the corresponding author/s.

## Author Contributions

LS: data curation, formal analysis, investigation, methodology, visualization, writing—original draft, and writing—review and editing. DW: data curation, visualization, writing—original draft, and writing—review and editing. ZY: formal analysis and writing—review and editing. CZ and AB: writing—review and editing. ZX: funding acquisition, project administration, and writing—review and editing. All authors contributed to the article and approved the submitted version.

## Conflict of Interest

The authors declare that the research was conducted in the absence of any commercial or financial relationships that could be construed as a potential conflict of interest.

## Publisher’s Note

All claims expressed in this article are solely those of the authors and do not necessarily represent those of their affiliated organizations, or those of the publisher, the editors and the reviewers. Any product that may be evaluated in this article, or claim that may be made by its manufacturer, is not guaranteed or endorsed by the publisher.
