# Supplementary material for: The FtcR-Like Protein ActR in Azorhizobium caulinodans ORS571 Is Involved in Bacterial Motility and Symbiosis With the Host Plant
Source: Front Microbiol. 2021 Nov 19;12:744268. doi: 10.3389/fmicb.2021.744268 (PMC8639532; doi:10.3389/fmicb.2021.744268)
Supplement: Supplementary file 1 [file Data_Sheet_1.pdf]

The FtcR-like protein ActR in *Azorhizobium caulinodans* ORS571 is involved in bacterial motility and symbiosis with the host plant

Li Sun[1†], Dandan Wang[1†], Zhiqiu Yin[1], Chengsheng Zhang[2*], Amber Bible[3], Zhihong Xie[1,4*]

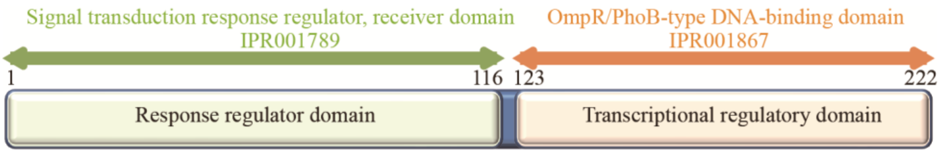

Supplementary Figure 1. Schematic representation of the ActR protein in the *A. caulinodans* ORS571 with the location of the domain. The domains predicted by InterPro annotation. IPR001789: Signal transduction response regulator, receiver domain; IPR001867: OmpR/PhoB-type DNA-binding domains are shown as boxes.

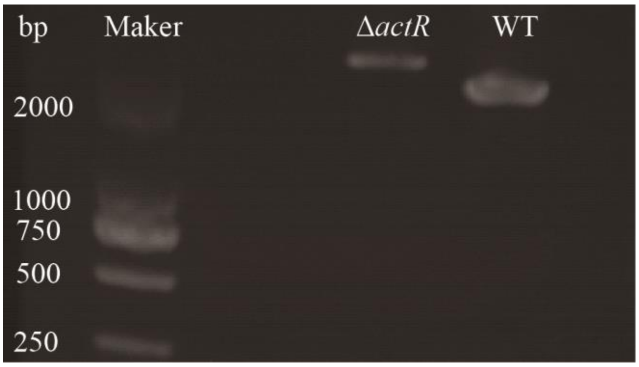

Supplementary Figure 2. PCR confirmation of the △*actR* mutant strain (Including maker (left), the genomic DNA of △*actR* mutant strain (middle), and the genomic DNA of WT (right)).

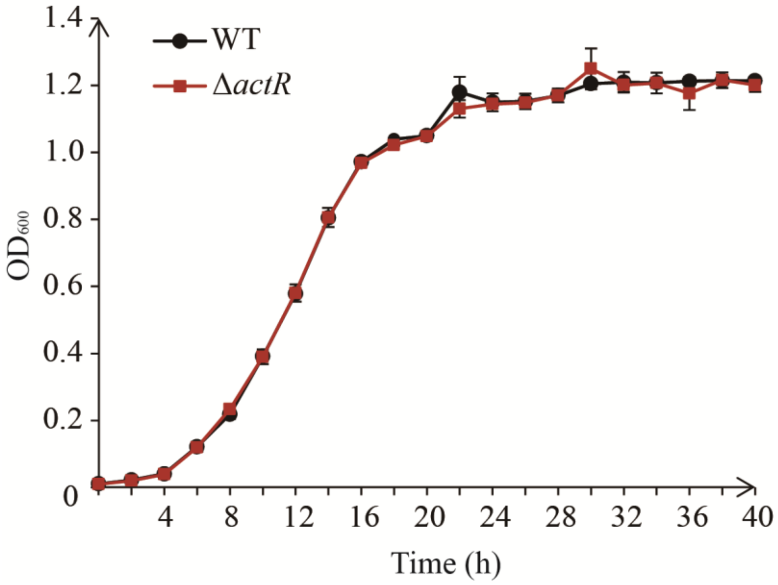

Supplementary Figure 3. Growth of the wild-type *A. caulinodans* ORS571 and △*actR* mutant strain in TY medium.
